# The dynamic of treatment-seeking in a community sample with obsessive-compulsive symptoms: A mixed method approach

**DOI:** 10.1371/journal.pone.0337010

**Published:** 2025-11-17

**Authors:** Winitra Kaewpila, Thanavadee Prachasan, Ratana Saipanish, Thanita Tantrarungroj, Fred Stevens

**Affiliations:** 1 Chakri Naruebodindra Medical Institute, Faculty of Medicine Ramathibodi Hospital, Mahidol University, SamutPrakan, Thailand; 2 Department of Psychiatry, Faculty of Medicine Ramathibodi Hospital, Mahidol University, Bangkok, Thailand; 3 School of Health Professions Education, Faculty of Health, Medicine and Life Sciences, Maastricht University, The Netherlands; University of Zanjan, IRAN, ISLAMIC REPUBLIC OF

## Abstract

**Objective:**

This mixed-method study aimed to investigate factors associated with treatment-seeking behaviors in people with obsessive-compulsive (OC) symptoms in the community and explore their experiences along the dynamic of treatment-seeking processes.

**Method:**

Eighty-one subjects with OC symptoms (27 treatment seekers and 54 non-seekers) completed online questionnaires about treatment history, symptom severity, and factors influencing treatment-seeking. The characteristics of treatment seekers and non-seekers were compared using Pearson’s Chi-square and independent T-tests. Qualitative data were derived from a subset of 26 participants undergoing a follow-up telephone interview and subsequently analyzed by thematic analysis.

**Results:**

Treatment seeking was associated with more severe overall OC and obsessive symptoms and more feeling out of control over the symptoms (p < .05). Qualitative analyses revealed three main themes of barriers (i.e., displacement of causation, perceived controllability, and thresholds to access treatment) intricately tied to the stages of help-seeking, from problem recognition to service utilization. The need to control was identified as a key determinant in shifting between the vicious OC-illusionary-control loop and the treatment-seeking-control loop along the dynamic of treatment-seeking processes.

**Conclusion:**

The symptom severity and feeling out of control are critical factors associated with treatment-seeking among people with OC symptoms in the community. Enhancing the feeling of control could be pivotal in promoting help-seeking behaviors in this population.

## Introduction

Obsessive-compulsive disorder (OCD) is a chronic, disabling psychiatric condition that affects approximately 2–3 percent of the global population, ranking as one of the 10 leading causes of disability worldwide [1]. Despite the availability of effective treatments, only around 30–40% of patients with OCD seek professional help [2]. Several large studies also showed that the average time to seek treatment is approximately 7 years after disturbing symptoms [3–5]. These pieces of evidence suggest that more insight into treatment-seeking decisions in this population is critically needed.

Numerous studies have sought to identify the factors that hinder or delay people with OCD from seeking treatment. Commonly cited barriers include specific characteristics of OCD symptoms, feelings of stigma or embarrassment, lack of knowledge and information about the disorder and available treatments, poor insight into the condition, and concerns related to treatment processes [2,6–8]. However, such findings are mainly derived from quantitative research in clinical cases of OCD already in treatment, limiting an in-depth understanding of the dynamic interplay of these factors on treatment-seeking behaviors and the generalizability of the findings to the undiagnosed population.

In the past two decades, OCD research has been extended beyond clinical diagnosis to subthreshold obsessive-compulsive (OC) symptoms to gain better insight across the OC spectrum. A German national health survey revealed that the 12-month prevalence of subthreshold OCD (i.e., fulfilling some but not all core DSM criteria) and OC symptoms (i.e., OC symptoms without fulfilling any core DSM criteria) were 4.5% and 8.3%, respectively [9]. Similarly, a US nationally representative survey estimated that over 25% of people in the community have OC symptoms at some point in their lifetime [10]. Furthermore, even below the diagnostic threshold, OC symptoms are also associated with more distress, psychiatric comorbidities, workplace problems, suicidality, and lower quality of life [9,11–13]. Indeed, more than 60% of patients with OCD report having subthreshold OC symptoms for at least one year before meeting the criteria for OCD, and almost half report a prolonged course of subclinical symptoms for five years or more [14]. Such empirical evidence supports the continuum nature of OC symptoms from subthreshold to clinical disorder, calling for more inclusive research into the subclinical end to guide future conceptualization of the condition and improve clinical care [9].

Therefore, in this study, we sought to identify people with OC symptoms in the community and used a mixed-method research design to gain more refined insight into the dynamic of treatment-seeking processes in this population. First, factors influencing symptom recognition, reasons for delayed treatment-seeking, and decision to get treatment were examined using a self-report questionnaire. Then, the dynamic process of treatment-seeking, from symptom recognition to service utilization, was further explored in a subset of samples using an in-depth telephone interview.

## Materials and methods

This research employed a sequential mixed-method design, combining an online survey with subsequent telephone interviews in a subgroup of participants. “Treatment-seeking” in the current study was restricted to formal help provided by healthcare professionals, including general practitioners, psychologists, psychiatrists, counselors, and other health practitioners [15]. The Institutional Review Board of the Faculty of Medicine Ramathibodi Hospital approved the study protocol (COA.MURA 2020/192).

### Participants and procedure

**Fig 1:**
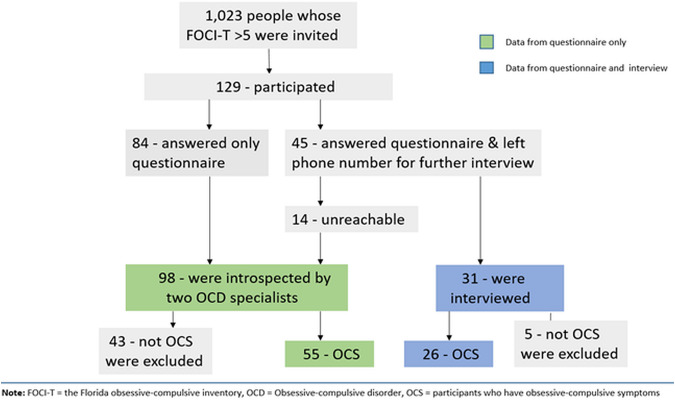
The flowchart of participant enrollment.

This research enrolled people who had completed the Florida Obsessive-Compulsive Inventory – Thai version (FOCI-T) [16] made freely accessible on the official website of the Department of Psychiatry, Faculty of Medicine Ramathibodi Hospital as a self-screening for OCD from 19 February 2020–13 March 2021. After completing the questionnaire, all respondents were asked to provide their phone numbers and/or e-mail addresses if they were willing to participate in OCD research. An invitation message to participate in this project was then sent to those whose FOCI-T score was above 5, at which cutoff the tool demonstrated 92% accuracy in identifying OCD cases [16]. After reading the participant information sheet, those who were aged 18 or above and online consented to participate in this study were asked to respond to a set of questionnaires inquiring about 1) biographic information, 2) obsessive-compulsive symptoms assessed by the Self-Report Version of the Yale-Brown Obsessive-Compulsive Severity Scale – Second Edition (YBOCS-II-SR) with an open question to describe their symptoms, 3) treatment history for OC symptoms, 4) factors influencing treatment-seeking assessed by the Interview on Help-Seeking Behavior (IH-S), and 5) willingness to be further interviewed over the telephone. To increase the specificity of identifying people with OC symptoms, two OCD specialists (RS and TT) independently considered the description of the symptoms provided by the participants. Those whose symptom description did not align with the characteristics of OC symptoms, such as lacking typical intrusive or ego-dystonic quality, were then excluded from further analyses. The final study sample consisted of 81 participants from 28 out of 77 provinces in Thailand. Of 31 participants verbally consenting to be interviewed, 5 lacked typical features of OC symptoms and were excluded, leaving 26 participants for the qualitative analysis. The summary of the participant enrollment is illustrated in Fig 1.

### Measures

#### Thai self-report version of the yale-brown obsessive-compulsive severity scale – Second edition (YBOCS-II-SR).

The YBOCS-II-SR, adapted from the Yale-Brown Obsessive-Compulsive Severity Scale – Second Edition (YBOCS-II) [17], was utilized to assess the severity of obsessive-compulsive symptoms. This self-report measure comprises five items evaluating time occupied, interference, distress, resistance, and degree of control for obsessive and compulsive symptoms. Each item was rated from 0 to 5, making up a total score ranging from 0 to 50. The higher the score, the more severe the symptoms. The internal consistencies of the Y-BOCS-II-SR total scores was excellent (α = 0.94). Confirmatory factor analysis showed adequate fit for obsession and compulsion factor models. The scale displayed strong and significant correlations with other established OCD measures, such as the Florida Obsessive-Compulsive Inventory (FOCI) and the Yale-Brown Obsessive-Compulsive Scale – Second Edition (YBOCS-II) [16] while presented weaker correlation with depression and quality of life measures, which implied good convergent and divergent validity. The Cronbach’s α of the scale in the current sample is 0.83.

#### Interview on Help-Seeking Behavior (IH-S).

The IH-S [2] was used to explore factors influencing treatment-seeking behaviors of individuals with OCD. The scale was adapted as a self-rated questionnaire and subsequently translated into Thai with permission from the author. It was used in the study about the factors associated with treatment-seeking among the obsessive-compulsive disorder (OCD) patients [18]. It comprises two main questions: 1) the duration from the first symptom until deciding to receive treatment, and 2) the initial realization that problems, thoughts, or behaviors are abnormal. Additionally, the IH-S includes three subordinate question sections: 1) the reasons for realizing the problems (6 items), 2) the reasons for delaying treatment-seeking (10 items), and 3) the reasons for seeking treatment (10 items). Participants indicated their responses as “yes” or “no” for each item.

### Telephone interview

The telephone interview was performed using a semi-structured interview by one of the OCD-specialized psychiatrists (RS, TT). After confirming consent to be interviewed, which was evidenced in voice recording, the interviewer asked the participants to describe their symptoms, perceived causes, and the impact of the symptoms on their lives. Then, the interviewer explored their coping with the symptoms, including treatment-seeking behaviors. For those who have received treatment, the timing from symptom recognition to treatment seeking, the decision to seek help, the source of help, and the outcome of help-seeking were further explored. For those who decided not to seek treatment, the interviewer asked for the reasons for such a decision, the plan to live with their symptoms, and the potential source of help if they were to receive it. Each interview lasted 30–40 minutes. All were recorded and transcribed verbatim.

### Data analysis

For quantitative data, participants’ characteristics and responses to the IH-S questionnaire were compared between treatment seekers and non-seekers. The independent t-tests and Pearson’s Chi-squared tests were used for continuous and categorical variables, respectively.

For qualitative data, the transcripts were independently analyzed by the three researchers (RS, TT, WK) using a thematic analysis approach [19]. Initial codes were generated based on the data guided by existing literature on treatment-seeking [20–21]. Next, axial coding was performed by grouping and searching for themes emerging from the codes and data. Finally, the three researchers reviewed and discussed the themes to reach a consensus and finalize the analyses.

## Results

### Participants’ characteristics

Demographic characteristics of the participants are shown in Table 1. Compared to non-seekers, treatment seekers comprised a lower percentage of women (p = .023) and were older on average (p = .033) (Table 1). Non-seekers tended to have a higher proportion of students and unemployed people (56%) compared to treatment seekers (33.3%) (p = .059). Moreover, the pattern of OC symptoms significantly differed between the two groups. While rumination/intrusive thought was more prevalent among treatment seekers (56%) than non-seekers (26%) (p = .009), checking was more prevalent among non-seekers (54%) than treatment seekers (26%) (p = .018). Obsession, as well as overall OC symptoms, was more severe among treatment seekers than non-seekers (p < .05) (Table 1).

**Table 1:** Participant characteristics.

**Characteristics**	**Total (n = 81)**	**Untreated (n = 54)**	**Treated (n= 27)**	**p-value**
Female, n (%)	63 (78)	46 (85)	17 (63)	.**023**
Age (year), *M* (SD)	25.5 (5.8)	24.5 (5.5)	27.4 (5.9)	.**033**
Education, n (%)				.606
Below Bachelor’s degree	24 (30)	15 (28)	9 (33)	
Bachelor’s degree or higher	57 (70)	39 (72)	18 (67)	
Marital status (single), n (%)	72 (89)	47 (87)	25 (93)	.666
Employment status, n (%)				.059
Employed	42 (52)	24 (44)	18 (67)	
Unemployed/student	39 (48)	30 (56)	9 (33)	
Symptoms, n (%)				
Checking	36 (44)	29 (54)	7 (26)	.**018**
Rumination/intrusive thoughts	29 (35)	14 (26)	15 (56)	.**009**
Contamination	16 (20)	9 (17)	7 (26)	.324
Symmetry/ordering	13 (16)	10 (19)	3 (11)	.392
YBOCS-II-SR scores, *M* (SD)				
Obsession	12.0 (4.2)	11.3 (3.9)	13.4 (4.5)	.**039**
Compulsion	11.2 (4.5)	10.7 (4.1)	12.3 (5.0)	.123
Total	23.2 (7.6)	22.0 (7.3)	25.7 (7.6)	.**038**
Symptom duration (months), *M* (SD)	71.8 (79.4)	61.7 (66.7)	91.9 (98.4)	.107
Duration from the first symptom to treatment (months), *M* (SD)			55.8 (67.8)	

Note: *M*, mean; SD = standard deviation; YBOCS-II-SR = Thai self-report version of the Yale-Brown Obsessive-Compulsive Scale – Second Edition. P-value < 0.05 is shown in bold.

### Responses to help-seeking questionnaire

Detailed responses to the IH-S questionnaire are presented in S1 Table. Around 60% of all participants already realized from the beginning that their symptoms were abnormal. The most prevalent reason for such realization was the interruption of the symptoms in their activities, reported by almost 90% in both groups. However, the inability to control the behaviors/thoughts was the only reason reported significantly more frequently among treatment seekers (85.2%) than non-seekers (63%) (p = .039). No significant differences in the reasons for delaying treatment were observed between the two groups. For reasons to seek treatment, the persistence of the symptoms with uncontrollability and the interference of the symptoms with activities were reported by almost all treatment seekers. Feeling sad, realizing the seriousness of the problem, and increasing disturbance were also common reasons, reported by more than 80% of the participants getting treatment.

### Qualitative findings

**Fig 2:**
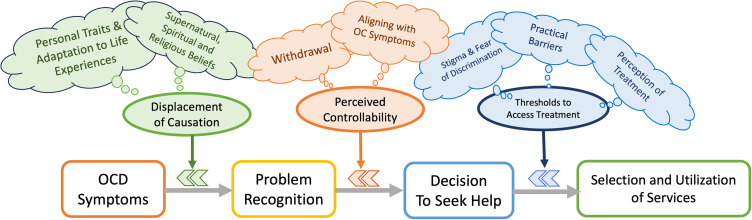
The treatment-seeking pathway and barriers.

Using thematic analysis, we identified several key themes that act as barriers preventing individuals with OCD symptoms from accessing and utilizing formal mental health services. These barriers are categorized into three main themes: 1) displacement of causation, 2) perceived controllability, and 3) thresholds to access treatment. As illustrated in Fig 2, these themes interact across the three stages of help-seeking (i.e., problem recognition, decision to seek help, and selection and utilization of services).

#### Displacement of causation.

From the beginning of OC symptoms, individuals often construct personal explanations for their experiences, significantly shaping their inclination away from recognizing them as a problem that needs professional help. Our research identified two primary perspectives among Thai participants that could hinder progression to the ‘problem recognition’ phase.

• **Personality Traits and Adaptation to Life Experiences**

Some individuals minimized their symptoms as mere quirks or transient worries, such as ‘just a thought’ or ‘just a little worry,’ or as aspects of their personality, like perfectionism or anxious traits. This minimization acts as a barrier to acknowledging their symptoms as indicative of a disorder, thereby delaying ‘problem recognition’.

*“I think it’s just a thought, not a disease. Maybe because I am more anxious than anyone else, not that I need to see a doctor…It’s not unsolvable, I thought it would be gone eventually.”* (female, 26, non-seeker, participant No.T2)

*“... I think it’s because of my perfectionism, it’s my personality. The situations [OC symptoms] those I have arose from perfectionists and perceived expectations from others (…)”* (male, 22, treatment seeker, participant No.T1)

Participants who linked their symptoms to previous traumatic events or life stressors were prone to justifying their compulsions as an adaptation.

*“...I’m not sure about the cause of this [OC symptoms], but there was an incident that made me compulsively check for safety. Many years ago, I caused a fire at my friend’s house. I lit a candle on and forgot to put it off, it then caused a fire in the bathroom and then burned the whole house....”* (female, 31, treatment seeker, participant No.R6)

• **Supernatural, Spiritual, and Religious Beliefs**

A portion of participants postponed consulting mental health experts, attributing their symptoms to supernatural forces best addressed through spiritual or religious practices.

*“...I ate beef, you see. My family worship Guan Yin (Goddess of mercy), so we do not eat beef. One time I ate beef meatballs which I thought it’s made from pork or chicken. It started from that and then many incidents followed…I was mad at…... [from holy objects and spirit], many bad things happened and I need to ask for forgiveness.”* (female, 36, treatment seeker, participant No.R7)

#### Perceived controllability.

Perceived controllability among individuals with obsessive-compulsive symptoms often surfaces as they begin to recognize their problem. During this process, many believe they can manage their symptoms independently, employing coping strategies like avoidance and self-monitoring. These strategies, though offering temporary relief, often obscure the need to seek professional help.

• **Perceived Controllability Through Withdrawal Approach**

Participants engaged in distraction and avoidance behaviors to circumvent OC symptom triggers. Although this approach temporarily provided a sense of control, it required sacrifices in significant areas of their lives and well-being. This fosters a deceptive sense of autonomy that deters professional help-seeking.

*“Participant: No, I haven’t used the public restroom at school since I ever remember, well only necessary like urgency*

*Interviewer: and other public restrooms outside of school?*

*Participant: No, I only wash my hands.*

*Interviewer: Does it affect your social life?*

*Participant: Yes, to some extent, when I go out with my friends, I’m afraid they will judge me.*

*Interviewer: So what did you do then in that situation?*

*Participant: I tried my best to hold it and come back home.*

*Interviewer: What about a trip with friends that you have to sleep over? is it affected?*

*Participant: Well,...I guess it has. I avoid not going, to some point they stop asking at all… (long silence).... “* (female, 18, non-seeker, participant No.R9)

• **Perceived Controllability Through Alignment With OC Symptoms**

Participants complied with their obsessions and compulsions as a method to alleviate distress. This coping mechanism, while offering momentary relief, led to the normalization of OC symptoms as a part of their life with a price of the detriment to their overall well-being.

*“…When my colleague touches my pen and my purse, I need to clean it. If I cannot do it my brain keeps repeating that I need to wash this and that and so… when I get home, no matter how late or how tired I was, I must clean it… Then it starts to feel something not right, not something a normal human should be doing. I used to think I’m just a super clean person, but then it was too much. I cannot explain how exhausting it was”* (female, 38, non-seeker, participant No.R21)

Both strategies foster a fleeting sense of autonomy, enticing individuals with OC symptoms. However, some participants developed depression, which significantly impaired their daily function, turning them towards seeking help.

*“…I went to see a psychiatrist when I was 14 or 15 years old…My parents took me to the hospital because of a drug overdose….I was so stressed from academics and family issues, and I changed school during that time…I continued seeing a psychiatrist because of my depression…If I only had intrusive thoughts, I wouldn’t have gone [to see a psychiatrist]…”* (female, 21, treatment seeker, participant No.R16)

*“...I was suffering… I couldn’t do anything… I didn’t know what happened to me... I just thought I was bad… I was wrong… It had been like this for two months before I went to see a doctor in tears... I didn’t know what to tell Doc what was wrong with me…but I knew I really needed help”* (female, 33, treatment seeker, participant No.R19)

#### Thresholds to access treatment.

The third theme, “Thresholds to Access Treatment,” builds on the first two themes: *Displacement of Causation* and *Perceived Controllability.* As individuals with obsessive-compulsive symptoms consider seeking help, they face barriers like fear of stigma, doubts about treatment, and uncertainty about symptom severity. These thresholds are difficult to cross, often pushing them back to self-management and reinforcing earlier patterns of attributing causes externally and trying to regain control independently.

• **Stigma and Fear of Discrimination**

Concerns over social stigma and exclusion lead many people with OC symptoms to hide their condition and be reluctant to seek professional help.

*“I am quite afraid of the idea of seeing a psychiatrist. Do these symptoms need psychiatric care? (said reluctantly) I am afraid of social exclusion. If I go, others will think I’m crazy, especially at work. There’s a joke when someone says something strange, they will say ‘go see a psychiatrist!’. One colleague who was diagnosed with depression was seen as ‘scary’ and others didn’t want to talk to her... I don’t want a negative reaction from my colleagues if they find out I went for treatment... so, if I can just hold on and keep it together, I would prefer that. Or if I need to go, it has to be in extremely bad condition that I could not live. And I would go to the internal medicine department first…”* (female, 26, non-seeker, participant No.T2)

• **Perception of** **Treatment**

Past experiences with the healthcare system deeply influence attitudes toward seeking future treatment. Having prior negative experiences, such as poor responses to treatment and medication side effects, can prime the person with the fear of potentially negative outcomes, resulting in continuing use of more familiar coping strategies rather than seeking formal help.

*“...the doctor gave me medication. But... uh... when I went to see the doctor, I didn’t feel much improvement. Just a bit better, but not quite enough. The doctor asked me to take the medication, and it felt like... you know, going to a barbershop, where they wash and dry your hair, finish up, and then give you some shampoo to take home. So, I went to the doctor, took the medication, and then went back home. I went there three times. At first, I felt okay, but the second and third times, I started to feel like it was probably a side effect of the medication, making me feel sleepy all day long. Then I felt nauseated and couldn’t focus at work...”* (female, 36, non-seeker, participant No.R15)

On the other hand, a lack of personal experience with psychiatric treatment often causes an individual to rely on hearsay, which may emphasize negative aspects of the healthcare system (e.g., long waiting times and poor service quality) or create a misconception about medical treatment, thus further discouraging help-seeking.

*“I read a lot about psychiatry practices. If it is not a private hospital, the service might not be good enough. It could even make patients worse, so I would not want to risk it. We have to pay a lot for it to get an Okay one (psychiatrist), the good one will have too many patients so they won’t have enough time for each of us. I thought it was really inaccessible so I wasn’t ready yet to go…”* (female, 25, non-seeker, participant No.R10)

*“...I heard that going to see a doctor, you will have to continue taking medications. I’m afraid that I would have kidney problems. If I take a lot of medications, then I would get a lot of physical diseases, right?”* (male, 21, non-seeker, participant No.R17)

• **Practical** **Barriers**

Practical issues, such as financial constraints and lack of information on accessing psychiatric services, stand as considerable obstacles. Financial independence and clear guidance on navigating the healthcare system are crucial for initiating treatment.

*“I want to see a psychiatrist but I don’t know where to start. Can I see a psychiatrist first or see GPs? No one gives me advice on who I should see? Where to go? and how? Should I walk in or do I have to make an appointment first?” (*female, 25, non-seeker, participant No.R22)

*“I don’t think it (psychiatric service) provides any help, they gave me medication and it was extremely expensive and it made me more stressed about the cost of medication instead, so I stopped going!”* (female, 23, treatment seeker, participant No.R20)

*“I’m afraid of the cost… I ensure that after I have graduated and worked, I think I should go and get treatment......I really don’t want to bother my parents about money.”* (male, 21, non-seeker, participant No.R17)

Support from family can greatly facilitate seeking effective treatment, especially among young people without financial independence.

*“Interviewer: In fact, you went to a psychiatrist at a young age.*

*Participant: Yes, I was in another province at that time. My mother always took me. We had to rent a van to visit the doctor quite often. After a week or two, we went to Bangkok and took a chartered van.”* (female, 36, treatment seeker, participant No.R7)

*“Mom brought me to see a psychiatrist at a clinic and receive the medications, but I didn’t get better...so my aunt who got treatment at XXX Hospital recommended me to see a doctor here”* (female, 32, treatment seeker, participant No.R11)

### Treatment-seeking dynamics

Sometimes the person can go back and forth along the three stages of help-seeking. For example, in participant No.R18, social support enabled partial problem recognition, pushing the individual toward seeking help; however, uncertainty about her condition and symptom fluctuation pulled her back from getting help.

*“Participant: They (friend) said, ‘You might have what was shown in that video.’*

*Interviewer: And that led you to look for more information based on their comments?*

*Participant: Yes, I wanted to know more because I started to think maybe I had it (the illness).*

*Interviewer: Have you ever seen a doctor?*

#### *Participant:* No

*Interviewer: Are you thinking about seeing a doctor now?*

*Participant: There was a time when I thought about seeing a psychiatrist.*

*Interviewer: Oh, why haven’t you gone yet?*

*Participant: No one has definitively told me that I have it.*

*Interviewer: So, you haven’t gone because you’re waiting for confirmation?*

*Participant: Yes, sometimes I get really frustrated, but then I forget about it because I’m generally in a good mood, so I didn’t go.*

*Interviewer: So, when things aren’t at their worst, the idea (of seeing the doctor) fades away?*

*Participant: Yes….“* (female, 36, non-seeker, participant No.R18)

Despite getting the information about the illness, this participant still identified herself with the symptoms and kept using situational withdrawal to manage her OC-related distress.

*“Participant: It’s like… I know I am like this, so I decided not to stay in a place where there are many people because I can’t control everybody. So, I’d rather be alone.*

*Interviewer: Ahh… So, you don’t think this is something abnormal, right?*

*Participant: It is rather a sin!*

*Interviewer: What do you mean?*

*Participant: I know I am abnormal, and not many people are like me.”*

(female, 36, non-seeker, Participant No.R18)

At the end of the interview, ambivalence to accept the symptoms as an illness and ignorance of where to seek help evidently emerged. These issues could be other vital barriers holding the person back from seeking treatment.

*“Interviewer: Okay! I’ll let you ask me now. Do you still have anything unclear? You could ask me then.*

*Participant: Do you think I have it (OCD)?*

*…*

*Participant: What do you think is the best way for me to get the right treatment?*

*Interviewer: Well, you might need to see a psychiatrist. They can give you guidance, and the important part is that they will follow up with you. That’s crucial, as they will give you tasks to work on and then track your progress. If medication is necessary, they will prescribe it, but if it’s not, you might not need to take any medication.*

*Participant: Is this kind of service available at a general hospital?”* (female, 36, non-seeker, participant No.R18)

## Discussion

This study used a mixed-method design to explore the dynamic of treatment-seeking in a community sample with OC symptoms. The quantitative investigation showed that participants who received psychiatric treatment had more severe overall and obsessive symptoms than their untreated counterparts. A more significant proportion of treatment seekers perceived their symptoms as uncontrollable compared to the untreated group. The qualitative investigation revealed three main themes of barriers (i.e., displacement of causation, perceived controllability, and thresholds to access treatment) intricately tied to the stages of treatment-seeking within the Thai sociocultural context.

Extending our investigation into subclinical and undiagnosed samples in the community, we confirmed the positive link between the severity of OC symptoms and help-seeking as previously reported [10,22]. Specifically, obsessive symptoms, but not compulsive symptoms, were significantly more severe among treatment seekers compared to non-seekers. Such an observation might reflect the anxiogenic nature of obsession that creates the necessity to seek treatment, contradictory to the anxiolytic nature of compulsion. Indeed, rumination/intrusive thought was more prevalent among treatment seekers, whereas checking was more prevalent among non-seekers in our sample. Even though an interrupting quality of the symptoms was the most rated reason for becoming aware of the problem in both groups, an inability to control thoughts/behaviors was the only reason reported significantly more often in treatment seekers than non-seekers. Such evidence underlined the significance of symptom severity, especially obsession, and feeling uncontrollable as critical features linked with treatment-seeking in this population.

Deepening the insight into the treatment-seeking dynamic within the Thai sociocultural context, we performed an in-depth telephone interview with a subset of participants. We identified three main themes of barriers resonating with the three stages of delay in help-seeking [23]. The three-stage model described sets of appraisal and decisional processes that delay symptom recognition, decision to seek help, and service utilization originally for physical illnesses [23]. This model was also subsequently applied to the process of seeking and obtaining help in children with emotional and behavioral disorders [24]. In line with the appraisal delay of the three-stage model, our first theme, displacement of causation, describes two forms of explanations commonly ascribed to OC symptoms that could delay symptom recognition. First, people with OC symptoms might perceive the symptoms as a part of their personal traits or adaptation to prior negative life experiences. Agreeing with personal values and concerns, such an explanation justifies the maintenance of OC-driven behaviors, underlying the hesitation to seek treatment as their sense of self is inseparable from their symptoms. Indeed, qualitative studies from the UK similarly revealed that patients with OCD and their family members often perceive OC symptoms as part of the person’s characteristics and identity, resulting in a belief in symptom permanence and lack of hope for curability [25,26]. The second form of explanation is attributing the symptoms to external forces, like supernatural power, spirituality, and religious beliefs, which could be particularly common in developing countries and among ethnic minorities [27,28]. This aligned with prior studies showing that the frame of reference for mental disorders among Thais is profoundly connected to traditional values and cultural and religious beliefs, especially in Buddhism [29–31]. While the concept of dukkha, viewing suffering as a part of all lives, could help promote acceptance, resilience, and emotional control of mental distress; the law of karma, viewing a person’s present and future life partly as consequences of the person’s past deeds, might make some people see their mental issues as personal shortcomings instead of curable health problems, precluding them from seeking professional help. [29–31]

The second theme derived from our qualitative analysis is perceived controllability. This theme aligns with the illness delay of the three-stage model [23], which describes the process of deciding whether the problem needs professional help. Perceived controllability highlights the role of compulsive and avoiding behaviors in temporarily relieving or preventing obsession-provoked anxiety, therefore providing an illusionary sense of control. Eventually, the person becomes dependent on avoidance and obsession-compulsion loops, deterring a decision to seek help for the price of functional impairment. Concordantly, evidence from a Brazilian cohort also showed that contamination/cleaning symptoms, which can be controlled by avoiding or compulsive cleaning behaviors, are associated with longer latency to treatment [32]. In our sample, a form of behavioral compulsions like checking was also more prevalent in the untreated group. Taken together, these pieces of evidence support the role of behavioral compulsions in providing an illusionary sense of control and delaying treatment seeking.

Aligning with the utilization delay of the three-stage model, our third main theme, thresholds to access treatment, comprises three subthemes of common barriers to service utilization for mental health issues. The first subtheme involves stigma and fear of discrimination, which have been consistently reported as key barriers to help-seeking for mental health problems across cultures and age groups [33–38]. This issue is critical in Thai society, where harmony, social connections, and group cohesion are common concerns [29]. The second subtheme covers practical barriers to treatment access, including ignorance of where to get psychiatric care, long waiting lists, and financial constraints. These issues are relevant to the perceived barriers (i.e., obstacles to performing a recommended health action) based on the health belief model (HBM) [39] and have also been consistently reported across the globe, even in high-income countries [28,40,41]. Moreover, the fact that the untreated participants in our sample tended to be less employed than treatment seekers implies the role of financial independence as an obstacle to seeking treatment. On the contrary, many participants reported receiving family support to facilitate access to effective therapy and retention in treatment, highlighting the role of social support in getting effective treatment. Finally, the third subtheme of barriers involves negative impressions of psychiatric services derived from personal experiences and opinions shared over the internet, partly reflecting the inadequacy of mental health services in Thailand. On top of that, during the treatment-seeking proceeds, the non-linearity of the three stages of help-seeking was evident. The non-sequential pattern is driven by the dynamic interplay between enablers and barriers which results in delaying treatment seeking. Despite different aspects of the barriers, the common issue underlying the three subthemes is fear of uncontrollable aspects of treatment-seeking within their social context and healthcare system.

**Fig 3:**
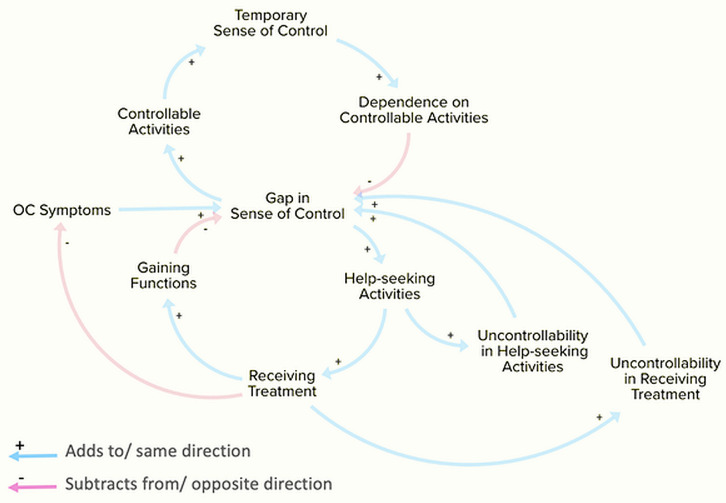
The causal loop diagram highlights that perceived control is central to whether individuals with OCD seek treatment.

Over the past two decades, control belief has gained more attention as one of the cognitive signatures for OCD, which could be a promising target for intervention. Experimental studies have consistently supported a desire to control and a sense of control as key distinctive features of OCD [42,43]. A recent systematic review also found that multiple aspects of control beliefs are associated with different OC symptoms [44]. Adding on existing evidence, our qualitative and qualitative findings similarly point to a critical role of control issues in the dynamic of treatment-seeking. As summarized in Fig 3, the causal loop starts with the person recognizing the uncontrollability of his/her thoughts and/or behaviors. The person might first try to make sense of their experiences by ascribing the symptoms to internal or external causes. While such a meaning-making effort could give them a partial sense of control, it could prevent them from searching for alternative explanations that lead to problem recognition and treatment-seeking. To tackle the residual feeling of control loss, the person could exploit situational avoidance and compulsive behaviors to restore a sense of control, albeit temporarily (Fig 3, the upper loop), or alternatively seek treatment (Fig 3, the lower loop). A trade-off between aligning with OC symptoms and the uncertainty and uncontrollability related to treatment-seeking must be made. Therefore, interventions that help people with OC symptoms become aware of the pathological nature of the symptoms and the illusionary sense of control provided by the OC loop and reduce the perceived obstacles associated with treatment-seeking could be a strategic tipping point to promote treatment-seeking behaviors, leading to functional restoration and improved quality of life in this population.

Notwithstanding, seeking professional help in real-world situations is generally far from ideal. Stigma, not knowing where to get help, long waiting lists, and financial constraints remain major barriers to treatment access [38,45]. Therefore, multifaceted interventions are required to overcome such hindrances.

First, providing educational resources to distinguish between normal behaviors and compulsive symptoms might help unveil the pathological nature of OC symptoms. As perceiving benefits of OCD treatment is an important predictor of help-seeking intention despite treatment barriers [46], successful stories from lived experiences might be particularly helpful for the person to identify with the case, externalize their symptoms, and become aware of an illusionary control provided by the OC loop, which would free the person from shame bound to the symptoms and promote treatment-seeking.

Second, to minimize negative perceptions and maximize the sense of control in treatment-seeking processes, practical information regarding treatment options, expected course of treatment, and different channels to access help should be provided in such a way that emphasizes the person’s autonomy in treatment decision and instills hope of regaining control over their functional lives [47].

Lastly, as the availability of provided service is a key enabling factor to assist people in using services according to Anderson’s behavioral model [48], self-help and technology-delivered interventions for OCD could be another promising strategy to cut through the shortage of mental health personnel, logistic and financial barriers to treatment, stigmatization, and fear of loss of autonomy [49–51], shifting the balance from the vicious OC-illusionary-control loop toward the treatment-seeking-control loop. Indeed, recent meta-analyses have shown that self-help interventions for OCD are both well-accepted and efficacious for symptom reduction despite a higher dropout rate compared to clinician-guided interventions [51,52]. As minimally assisted self-help intervention has been shown to increase treatment adherence, providing such an option might be another solution to cross over barriers to treatment for this population.

### Strength and limitations

The strengths of our study include the use of a mixed-method design to elucidate the dynamic process of treatment-seeking in the Thai sociocultural context, which might be partly generalized to other low-to-middle-income countries. Besides, our attempt to seek subthreshold and undiagnosed cases in the community reveals similar issues as previously reported in clinical samples, providing a more complete picture of treatment-seeking across the OC spectrum. The insight derived from this study might be useful for the mental health personnel in designing the inclusive strategies for promoting help-seeking behaviors in people with OC symptoms in the community.

Nevertheless, the interpretation of our study should be considered in the light of limitations. First, the sample size of 81 participants is relatively small, limiting the generalizability of the study’s findings. However, the geographic spread, with participants from 28 of Thailand’s 77 provinces, supports heterogeneity and enhances the transferability of findings. Furthermore, in keeping with the study’s mixed methods design, the qualitative component was not intended to produce statistically representative results, but to explore patterns, meanings, and contextual factors that may inform future research or policy. Second, all participants in this study were volunteers; self-selection bias may arise in the recruitment process. In other words, treatment-seeking attitudes among those voluntarily participating in research may differ from the general population. Moreover, we used a screening questionnaire complemented by self-described symptoms and a telephone interview to identify participants with OC symptoms; therefore, an unintentional reporting bias might be created [53], and a definite clinical diagnosis cannot be made. However, it may reflect real-world pathways through which individuals with obsessive-compulsive symptoms engage with mental health resources, lending ecological validity to the findings. Third, our study did not systematically assess comorbid illnesses unless the participants mentioned them specifically during the interview. Consequently, we might have missed other comorbidity-related factors that could interfere with treatment-seeking. Forth, as we enrolled participants via online screening, our findings might not be generalized to those with poor technological literacy or lack of access to the internet. Specifically, the majority of our samples are female, aged around 20–30, unmarried, and having at least a bachelor’s degree. Therefore, future studies should also employ a non-technological-based approach to ensure the inclusion of a wide variety of populations, including males, the older generation, and less educated participants, to confirm our findings.

## Conclusion

The overall symptom severity, especially obsessional domain, and feeling out of control are critical factors associated with treatment-seeking among people with OC symptoms in the community, aligning with evidence from the clinical population. Qualitative data underscore the pivotal role of controllability along all stages of treatment-seeking, from symptom recognition to service utilization in this population. The interventions that help people with OC symptoms become aware of the pathological nature of the symptoms and the illusionary sense of control provided by behavioral avoidance and compulsion and reduce the perceived obstacles associated with treatment-seeking could be a strategic tipping point to promote treatment-seeking behaviors, leading to functional restoration and improved quality of life in this population.

## Supporting information

S1 Table The Interview of Help-Seeking (IH-S) – Numbers and percentages of reported from 81 participants. (DOCX)
