# Correction: The dynamic of treatment-seeking in a community sample with obsessive-compulsive symptoms: A mixed method approach

**DOI:** 10.1371/journal.pone.0339702

**Published:** 2025-12-26

**Authors:** Winitra Kaewpila, Thanavadee Prachason, Ratana Saipanish, Thanita Tantrarungroj, Fred Stevens

The second author’s name is spelled incorrectly. The correct name is: Thanavadee Prachason. The correct citation is: Kaewpila W, Prachason T, Saipanish R, Tantrarungroj T, Stevens F (2025) The dynamic of treatment-seeking in a community sample with obsessive-compulsive symptoms: A mixed method approach. PLoS One 20(11): e0337010. https://doi.org/10.1371/journal.pone.0337010.
