# Supplementary material for: The dynamic of treatment-seeking in a community sample with obsessive-compulsive symptoms: A mixed method approach
Source: PLoS One. 2025 Nov 17;20(11):e0337010. doi: 10.1371/journal.pone.0337010 (PMC12622799; doi:10.1371/journal.pone.0337010)
Supplement: S1 Table — (DOCX) [file pone.0337010.s001.docx]

**S1 Table. The Interview of Help-Seeking (IH-S) – Numbers and percentages of reported from 81 participants**

|  | **All**  **(n = 81)** | **Untreated**  **(n =54)** | **Treated**  **(n= 27)** | **p-value** |
| --- | --- | --- | --- | --- |
| **Duration from the first symptom until deciding to receive treatment (months)** |  | - | 55.8 ± 67.8 | - |
| **Did you realize from the beginning that your problems/thoughts/behaviors were not normal?**  **- Number of “Yes” answer** | 48 (59.3%) | 33 (61.1%) | 15 (55.6%) | 0.631 |
| **Section I. How did you realize you had a problem?** | | | |  |
| - It interrupted what I was doing | 72 (88.9%) | 48 (88.9%) | 24 (89.7%) | 1.000 |
| - I noticed changes in my behavior | 63 (77.8%) | 40 (74.1%) | 23 (85.2%) | 0.257 |
| - I felt sad | 60 (74.1%) | 37 (68.5%) | 23 (85.2%) | 0.107 |
| - Someone told me that my behaviors (and/or concerns) were unusual or strange | 59 (72.8%) | 37 (68.5%) | 22 (81.5%) | 0.216 |
| - I have information about the disorder | 59 (72.8%) | 37 (68.5%) | 22 (81.5%) | 0.216 |
| - I couldn’t control (my behavior and/or thoughts) | 57 (70.4%) | 34 (63.0%) | 23 (85.2%) | 0.039* |
| **Section II. Why did you delay in seeking treatment for your problem?** | | | |  |
| - I believed my behaviors and/or thoughts were not serious | 59 (72.8%) | 41 (75.9%) | 18 (66.7%) | 0.826 |
| - I was convinced that the problem was temporary (it would pass over the time) | 50 (61.7%) | 34 (63.0%) | 16 (59.3%) | 0.361 |
| - I was afraid (of the thought contents) | 49 (60.5%) | 30 (55.6%) | 19 (70.4%) | 0.118 |
| - I felt I could control the problem | 48 (59.3%) | 34 (63.0%) | 14 (51.9%) | 0.297 |
| - I felt ashamed by the thought contents | 41 (50.6%) | 26 (48.1%) | 15 (55.6%) | 0.265 |
| - I feared that if I disclosed the thoughts to someone, the thoughts could come true | 40 (49.4%) | 28 (51.9%) | 12 (44.4%) | 0.325 |
| - I feared being considered a mentally ill person | 37 (45.7%) | 23 (42.6%) | 14 (51.9%) | 0.234 |
| - I was afraid someone would tell me I was a bad person for having these thoughts | 35 (43.2%) | 22 (40.7%) | 13 (48.1%) | 0.269 |
| - I thought it was not a problem requiring professional help or treatment | 34 (42.0%) | 21 (38.9%) | 13 (48.1%) | 0.235 |
| - The problem did not interfere in my daily activities | 18 (22.2%) | 15 (27.8%) | 3 (11.1%) | 0.098 |
| **Section III. Why did you seek treatment for your problem?** | | | |  |
| - The problem didn’t disappear; I couldn’t control it |  | - | 26 (96.3%) | - |
| - The problem (thoughts and/or behaviors) interfered with what I was doing |  | - | 25 (92.6%) | - |
| - I felt sad |  | - | 23 (85.2%) | - |
| - I thought I had a serious problem (an illness) |  | - | 22 (81.5%) | - |
| - The problem (thoughts and/or behaviors) became more and more disturbing |  | - | 22 (81.5%) | - |
| - I was afraid of what was happening to me |  | - | 21 (77.8%) | - |
| - The problem (thoughts and/or behaviors) became more frequent |  | - | 21 (77.8%) | - |
| - Someone advised me to seek treatment |  | - | 21 (77.8%) | - |
| - I believed that my thoughts could come true |  | - | 15 (55.6%) | - |
| - I thought I was a bad person for having these thoughts |  | - | 11 (40.7%) | - |

*p<0.05
